# Innovations and Emerging Trends in Prostate Cancer Management: A Literature Review

**DOI:** 10.7759/cureus.73128

**Published:** 2024-11-06

**Authors:** Nazeer Ibraheem, Momen Abdelglil, Andrew Wanees, Ahmed M Aosmali, M Hasaan Shahid, Reda H Mithany

**Affiliations:** 1 Urology, The Royal Wolverhampton NHS Trust New Cross Hospital, Wolverhampton, GBR; 2 Pediatric Surgery, Mansoura University Children Hospital, Mansoura, EGY; 3 General Surgery, Ain Shams University Hospitals, Cairo, EGY; 4 Trauma and Emergency Surgery, King’s College Hospital NHS Foundation Trust, London, GBR; 5 Surgery, Glangwili General Hospital, Carmarthen, GBR; 6 Colorectal Surgery, Torbay and South Devon NHS Foundation Trust, Torquay, GBR

**Keywords:** chemotherapy, hormonal treatment, immunotherapy, precision medicine, prostate cancer (pc), radiotherapy, targeted therapy

## Abstract

Prostate cancer (PC) is considered the second most diagnosed cancer in men worldwide. It remains a leading cause of cancer-related death. Recently, many modalities have been discovered and used in the diagnosis and management of PC, with the incorporation of many treatment options such as hormonal therapy, chemotherapy, targeted therapies, immunotherapy, and precision medicine. Robotics and artificial intelligence (AI) have further modified the diagnosis and management of PCs, improving the diagnosis accuracy and disease progression. This comprehensive review offers an in-depth exploration of the historical modalities of treatments, an evaluation of current therapeutic techniques, a discussion of the use of robotic surgery and AI, and an examination of ongoing clinical trials and emerging procedures. Additionally, this review also covers the challenges. By inspecting these aspects, the review may provide valuable information regarding future research and clinical practice directions in PC treatment, contributing to a thorough understanding of the complex and emerging context of PC management.

## Introduction and background

Prostate cancer (PC) is the second most common cause of cancer-related deaths among men globally, necessitating the urgent need for awareness and prevention strategies. Around 1.4 million new cases are diagnosed yearly, predominantly affecting the age group 65 and above. Risk factors such as age, family history, race, and diet are important in the disease course and progression. PC continues to contribute to a substantial disease challenge worldwide. However, advances in early diagnosis through prostate-specific antigen (PSA) screening involve ongoing efforts in research and treatment innovation [1,2].

Breakthroughs in PC treatment have improved over the past few years. Historically, surgery and radiation therapy were the cornerstones of treatment, but these approaches were often limited in effectiveness, especially for metastatic cases. As our understanding of the molecular and genetic underpinnings of PC deepened, new treatment options, such as androgen receptor (AR) inhibitors and immunotherapy, emerged. Moreover, the use of robotics and artificial intelligence (AI) in PC treatment has enhanced surgical precision, diagnosis, and individualized treatment. These innovations have not only improved the disease outcome but have also reduced treatment-related drawbacks, offering patients a better life [3,4]. Although novel hormone therapies such as abiraterone and enzalutamide have shown promise, many patients eventually develop resistance to these treatments, necessitating the need for more durable therapeutic options [5]. This review aims to explore the new advances in the management of PC.

## Review

Historical review

The modalities of PC have changed significantly. Over the past century, there have been three chairpersons of urology at Johns Hopkins. The first, Dr. Hugh Hampton Young, is widely regarded as the founder of modern urology. In the 1940s, many changes in surgical intervention, such as perineal and retropubic prostatectomy, offered the ability to manage early PC effectively [6]. This procedure involves the removal of the entire gland and the surrounding lymph node. However, this procedure resulted in many complications, including urinary incontinence and erectile dysfunction, which negatively affected the quality of life for many patients [7].

Radiation therapy (RT) was introduced as a treatment for PC in the mid-20th century and emerged as an alternative modality for surgery. Conventional external-beam RT can destroy cancerous cells using high-beam energy radiation. However, early radiation techniques lacked precision, often causing damage to the surrounding organs and leading to comparable side effects as surgery, limiting its effectiveness in curing cancer with reduction of side effects [8].

**Figure 1:**
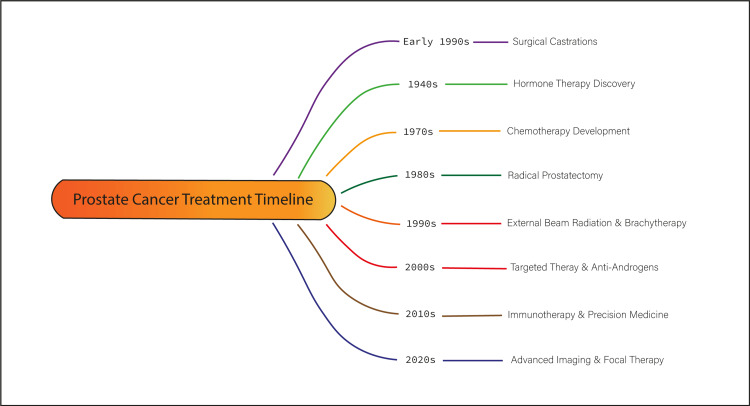
Prostate cancer treatment timeline. Image credit: Momen Abdelglil.

**Figure 2:**
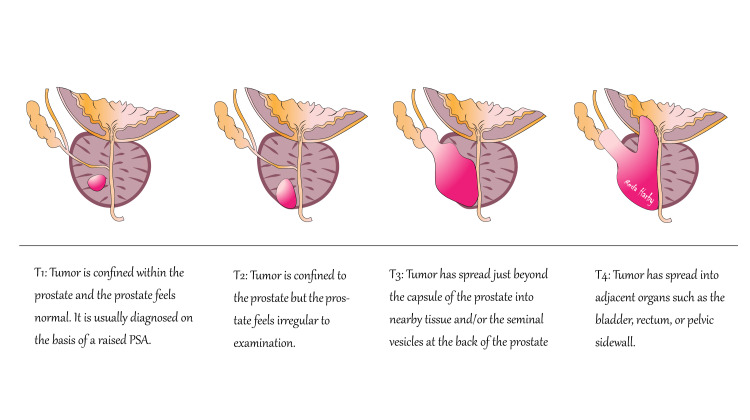
TNM staging of prostate cancer. Image credit: Reda Harby.

Both radical prostatectomy and early use of RT demonstrated limitations in long-term outcomes. For many patients, the treatments resulted in chronic side effects that could persist for years or even for life. The lack of precision in both surgical and radiological intervention may increase the risk of cancer recurrence [9]. Researchers have been seeking to improve the effectiveness of PC treatment. While surgery and radiation remained mainstays, their limitations spurred the development of less invasive and more focused therapies aimed at reducing side effects [7] (Figures 1, 2).

Use of artificial intelligence in the management of prostate cancer

AI can enhance the imaging techniques used in the diagnosis or staging of PCs. Machine learning algorithms can analyze MRI and positron emission tomography scans to detect prostate tumors with higher sensitivity and specificity. AI machines can detect subtle patterns and anomalies that may be overlooked by human observers, leading to more accurate diagnoses. For instance, convolutional neural networks (CNNs) have been applied to multiparametric MRI systems to improve the detection of clinically significant PC. This enhancement in diagnostic accuracy facilitates timely treatment interventions and guides the selection of appropriate therapeutic strategies [10].

**Figure 3:**
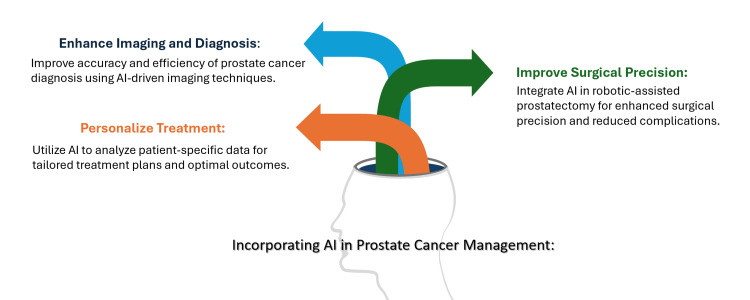
How to incorporate artificial intelligence (AI) in prostate cancer management. Image credit: Reda Harby.

AI has significantly improved RT planning in PC treatment by enhancing its precision. RT requires meticulous targeting of malignant tissues to save the surrounding healthy organs. AI algorithms, particularly deep learning models, can help in the differentiation between malignant and non-malignant tissues. This automation can reduce interobserver variability and improve the accuracy of contouring, which is crucial for accurate RT. Further, recent studies have demonstrated that AI-assisted segmentation achieves performance comparable to that of expert clinicians. Moreover, AI models optimize radiation dose distributions by matching patient-specific anatomical and physiological characteristics to improve therapeutic outcomes and decrease adverse effects [11] (Figure 3).

Artificial Intelligence-Enhanced Imaging and Diagnosis in Prostate Cancer

AI has improved PC diagnosis, offering improvements in its accuracy and efficiency. Traditionally, diagnosing PC has relied on invasive methods such as biopsies and imaging techniques such as MRI and CT. However, these methods often suffer from significant drawbacks, including the need for specialized expertise and high variability in interpretation. AI applications in PC diagnosis, particularly in imaging, have shown the ability to address these challenges by enhancing the detection of suspicious lesions and reducing unnecessary biopsies. AI-driven models, such as CNNs, have been trained to process vast amounts of radiological data, distinguishing between malignant and benign lesions more accurately than classic methods​ [12]. In different studies, AI models have even demonstrated the power to automatically assign Gleason scores, a key prognostic indicator, with a high degree of accuracy, reducing the reliance on subjective human results. This automation not only enhances the results but also speeds up the process, which aids in earlier intervention and improved prognosis [13-15].

Artificial Intelligence in Risk Stratification and Prognostic Biomarkers

Risk stratification in PC is a condemnatory part of determining treatment approaches and predicting disease progression. However, classic risk stratification methods such as the National Comprehensive Cancer Network protocols have not shown the ability and accuracy to predict patient outcomes. AI presents a solution to these limitations by using various data types, such as clinical, genetic, and imaging data, into preformed models that can offer more precise results. Machine learning algorithms were created to predict the incidence of biochemical recurrence or metastasis based on patient data. For instance, studies have shown that learning models can predict recurrence or disease progression by analyzing multiparametric MRI data in conjunction with histopathology data [12,16]. Moreover, the ability of AI to summarize and inspect complex data forms allows the identification of novel prognostic factors that can predict patient outcomes and guide individualized diagnostic and treatment strategies. This level of precision is important for distinguishing between cases that need advanced treatment and those that can safely opt for active surveillance [17].

Artificial Intelligence-Driven Treatment Personalization

AI can analyze patient-specific data to predict types of suitable therapy, offering individualized treatment protocols that are tailored to individual’s current situation. One of the most promising uses of AI in this area is its ability to predict the patient response to androgen deprivation therapy (ADT) or RT. By investigating genetic, imaging, and clinical parameters, AI can help clinicians determine whether a patient will benefit from a specific treatment or if an alternative procedure should be used for treatment. Furthermore, AI models have been used in clinical protocols to assist in RT agreements. These models intensify treatment modalities by predicting radiation sensitivity and ensuring that therapy targets cancer cells while sparing other normal cells. This level of precision is especially important in PC, where the adverse effects of the treatment can affect a patient’s quality of life [18,19].

Artificial Intelligence-Assisted Robotic Surgery in Prostatectomy

AI plays an important role in robotic-assisted prostatectomy procedures to enhance surgical accuracy and results. AI algorithms can help surgeons by providing real-time feedback, predicting anatomical structures, and identifying the best surgical approaches. This modality is helpful in radical prostatectomy nerve-sparing procedure, where preserving the neurovascular bundles is important for postoperative sexual and urinary functions. Recent studies have shown that AI-enhanced robotic systems reduce operative times and improve outcomes for patients undergoing radical prostatectomy. The combination of AI and robotics-assisted laparoscopic surgeries represents a great breakthrough in minimally invasive surgical operations for PCs [20-22].

**Figure 4:**
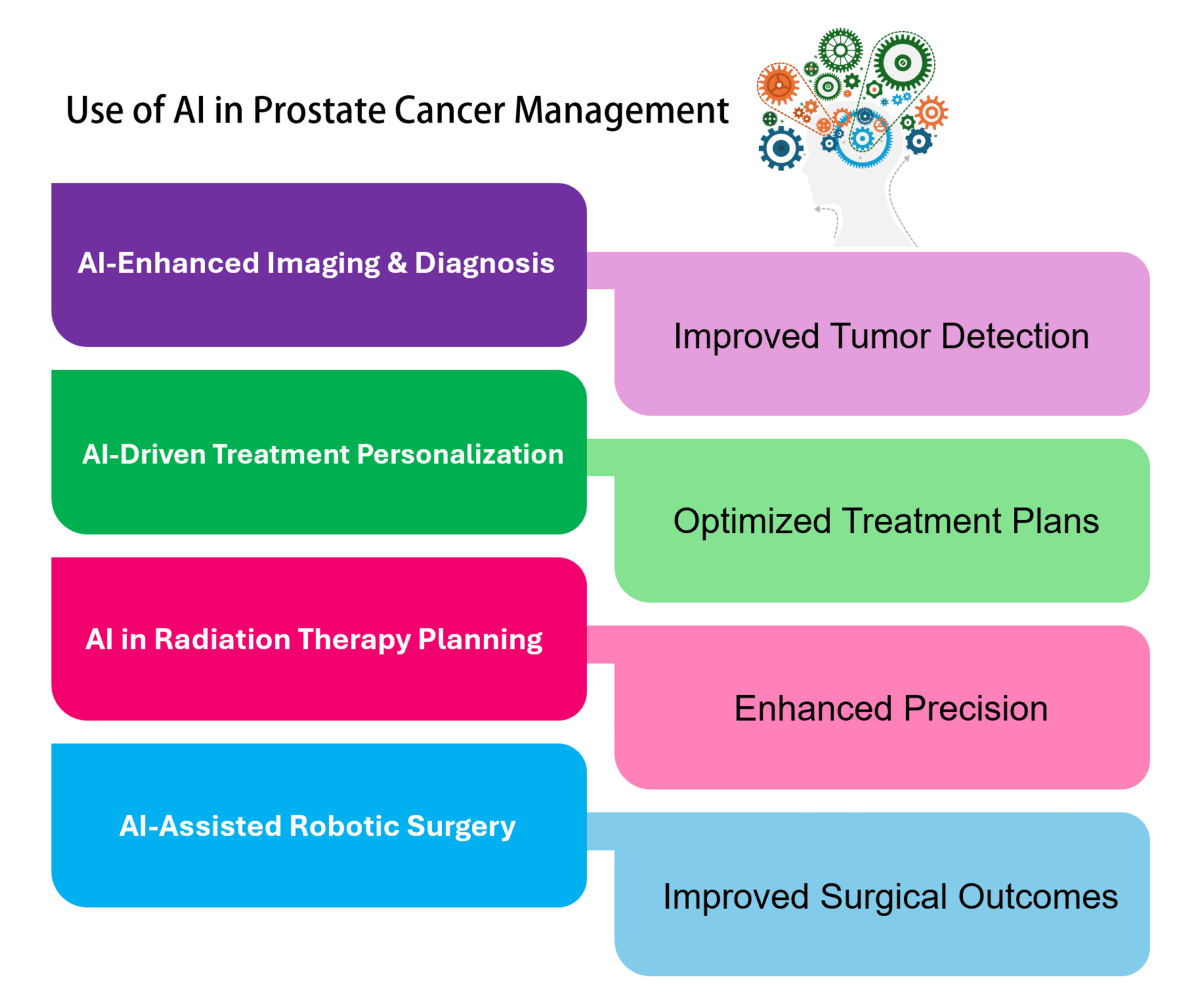
Summary of use of artificial intelligence (AI) in prostate cancer management. Image credit: Reda Harby.

The development and application of AI-based techniques in robotic surgery represent an important innovative approach to improving a patient’s prognosis. AI systems can employ a neural network to predict expected intraoperative bleeding during robot-assisted radical prostatectomy, showing high accuracy with 98% true-positive results and the ability to foresee bleeding occurrence. This innovative approach offers a chance to improve patient safety by providing early warnings to surgeons, thereby allowing them to take pre-emptive measures to avoid these problems. While the current system has some drawbacks, such as the need for a larger dataset and faster processing times, ongoing research and development are expected to solve these challenges. AI technologies can be used in the medical field, particularly in enhancing the safety and efficiency of complex procedures such as robotic surgeries [23-25] (Figure 4).

Advances of immunotherapy in prostate cancer treatment

Traditional treatment options, such as ADT, have been somewhat successful in managing the early stages of PC, but metastatic castration-resistant prostate cancer (mCRPC) remains a difficult condition. Recently, immunotherapy has emerged as a promising avenue for cancer treatment, with approaches such as immune checkpoint inhibitors (ICIs), chimeric antigen receptor (CAR) T-cell therapy, and therapeutic vaccines being accepted with great effects [26].

Sipuleucel-T, the first FDA-approved therapeutic cancer vaccine for PC, has demonstrated several benefits. It works by enhancing the ability of the patient’s immune system to recognize and attack PC cells. In clinical trials, Sipuleucel-T prolonged overall survival by 4.1 months in patients with mCRPC. Another vaccine, PROSTVAC, has also been tested for its potential to stimulate the immune system against PC. Although early-phase trials showed promising results, a phase III trial failed to demonstrate significant overall survival benefits. Nonetheless, ongoing research into combination therapies, such as Sipuleucel-T or PROSTVAC with ICIs, holds promise for enhancing the results of vaccine-based immunotherapy​ [27].

One of the key challenges in PC immunotherapy is the tumor’s immunosuppressive media. Unlike “hot” tumors such as melanoma, which contain a high density of tumor-infiltrating lymphocytes (TILs) and exhibit strong responses to ICIs, PC is typically classified as a “cold” tumor. It lacks significant immune cell infiltration and exhibits low levels of tumor-associated antigens, making it difficult for the immune system to detect or attack the cancer cells. Studies have shown that the immunosuppressive microenvironment of PC is characterized by the presence of immunosuppressive cells, such as tumor-associated macrophages and regulatory T cells, which suppress the activity of effector T cells and prevent a good immune response. This presents a significant barrier to the success of immunotherapy, and recent research has focused on strategies to overcome these problems [28].

Another development in PC immunotherapy is the use of CAR T-cell treatment, which involves tailoring a patient’s T cells to recognize and combat cancer cells. While CAR T-cell therapy has shown a good prognosis in treating blood cancers, its application in solid tumors such as PC is difficult. One of the main barriers is the dense stromal tissue, which surrounds the prostate tumors in addition to a limitation in the ability of CAR T cells to go through its process. Recent modifications in CAR T-cell design, such as the incorporation of mechanisms to target the tumor microenvironment and intensify T-cell trafficking, have shown potential in overcoming these obstacles. For instance, researchers are testing the use of CAR T cells that target prostate-specific membrane antigen (PSMA), a protein present on PC cells, in combination with other immunotherapies to improve their effect and target the best results in fighting PC [26,27,29].

Novel androgen receptor-targeted therapies

ADT is considered a keystone for the treatment of metastatic PC. However, long-term ADT often leads to resistance by the emergence of castration-resistant prostate cancer. Novel AR-targeted therapies can inhibit this resistance by disrupting AR signaling. Recently, proteolysis-targeting chimeras (PROTACs), which promote AR degradation, have shown great results. These therapies use small molecules to degrade the AR and decrease its cancer-promoting activities, providing an alternative to traditional inhibitors that focus solely on blocking AR signaling [30].

Another innovative approach involves heat shock proteins (HSPs), which stabilize ARs, allowing them to promote tumor growth. Targeting HSPs, particularly HSP90, can effectively reduce AR activity by degrading AR splice variants that contribute to therapy resistance. Drugs such as onalespib have been tested for their ability to inhibit HSP90, preventing AR splice variant expression and slowing cancer progression. However, despite the potential of HSP inhibitors, clinical trials have yet to yield consistent, widespread success, with challenges remaining in effectively translating preclinical findings into clinical protocols [30-35].

Despite these advancements, challenges still exist in optimizing AR-targeted therapies. Many patients continue to experience disease progression due to persistent AR signaling through alternative pathways or the emergence of AR splice variants such as AR-V7, which lacks the ligand-binding domain targeted by traditional therapies. Researchers are testing new ways to inhibit AR-V7, with promising preclinical data suggesting that targeting the CLK2/SRSF9 splicing axis may offer a solution. This approach aims to decrease AR-V7 expression and resensitize resistant cancer cells to AR-targeted therapies [30].

Advances in radiotherapy for prostate cancer

Over the past few years, there have been significant innovations aimed at enhancing the precision of radiotherapy for the treatment of PC, reducing exposure to surrounding healthy tissues, and improving treatment outcomes. The primary objective of radiotherapy is to deliver high-dose radiation to tumor sites while limiting damage to nearby organs, such as the bladder and rectum, which can reduce the risk of complications [36].

Over the years, advancements in radiotherapy modalities have significantly changed the precision of treatment, reducing side effects and enhancing outcomes. One of the major advances is stereotactic body radiation therapy (SBRT) which allows for high doses during radiotherapy to be delivered in just a few sessions. Compared to classic radiotherapy, which typically requires 20-40 sessions, SBRT completes treatment in fewer fractions. Clinical trials, such as the PACE-B study, have revealed that SBRT is similar to classic fractionation, showing a high incidence of targeting the disease with a 95.8% five-year survival rate for patients with intermediate-risk localized PC [37].

Another innovation is intensity-modulated radiotherapy (IMRT), which regulates the radiation beam intensity to deliver higher doses of radiation to the cancer cells while sparing the surrounding normal tissues, particularly the bladder and rectum. IMRT has become the standard of best practice for PC, allowing for more accurate targeting of the tumor and reducing adverse effects. Studies have shown that IMRT can lead to better long-term tumor progression and a reduction in side effects compared to older modalities. This is especially good for patients whose risk stratification is high-risk localized PC and who require higher doses for effective tumor control [38,39].

Proton beam therapy (PBT) offers another promising development, using protons instead of traditional photons to deliver radiation. This technique helps in the accurate spontaneous delivery of high-energy radiation with less exit dose, potentially minimizing the risk of long-term damage to surrounding healthy tissues. However, while proton therapy shows theoretical advantages, its clinical superiority over IMRT is still being discussed. Some studies suggest that PBT may reduce adverse effects, but there is not yet an absolute indication to support its widespread use over more established methods such as IMRT [40,41].

Lutetium-177-PSMA-617 is a breakthrough radioligand therapy that has been examined as a promising treatment for mCRPC, which is a condition signalized by the progression of PC despite hormone therapy and chemotherapy. This innovative therapy targets PSMA, which appears in PC cells, allowing for targeted delivery of beta-particle radiation directly to malignant cells. The efficacy of Lutetium-177-PSMA-617 was primarily demonstrated in the VISION trial, a pivotal phase 3 study that enrolled patients with mCRPC who had previously undergone at least one AR signaling inhibitor and one or two taxane-based chemotherapy regimens. In this trial, 831 patients were randomized to receive either Lutetium-177-PSMA-617 combined with standard care or standard care alone. The results indicated a significant improvement in both imaging-based progression-free survival (8.7 months vs. 3.4 months) and overall survival (15.3 months vs. 11.3 months) rates for those receiving the radioligand therapy [42].

Furthermore, the treatment was generally well tolerated, although there was a higher incidence of grade 3 or higher adverse events compared to classic care alone (52.7% vs. 38.0%). Importantly, quality of life metrics was not adversely affected by the treatment, suggesting that Lutetium-177-PSMA-617 can provide clinical benefits without significantly compromising individual well-being [43,44]. Real-world evidence also supports the effect of this therapy, showing that over 50% of patients experienced a significant decrease in PSA levels following treatment, further validating its role in managing advanced PC [43]. As such, Lutetium-177-PSMA-617 can be a significant advancement in the therapeutic landscape for mCRPC, offering hope for improved survival rates and quality of life for patients facing this challenging disease [45,46].

A precision medicine approach

Poly (ADP-ribose) polymerase (PARP) inhibitors (PARPi) emerged as an important advancement in the treatment of PC, particularly in cases characterized by DNA repair deficiencies. PARPi function by exploiting the concept of synthetic lethality, particularly in cancer cells that have compromised homologous recombination repair (HRR) pathways. Approximately 20% to 25% of patients with mCRPC harbor mutations in genes associated with DNA damage repair, such as *BRCA1* and *BRCA2*. These mutations render tumor cells unable to effectively fix double-strand breaks (DSBs) in DNA, making them particularly susceptible to PARPi. By inhibiting PARP activity, these drugs prevent the repair of single-strand breaks (SSBs), which subsequently leads to the accumulation of DSBs and ultimately results in cell death [47,48].

The dual mechanisms through which PARPi works include competitive inhibition of PARP’s catalytic activity and trapping PARP on damaged DNA. This trapping effect prevents the release of PARP from the DNA strand, further complicating the repair process and enhancing cytotoxicity in HRR-deficient cells. This mechanism is particularly relevant for tumors under the classification of *BRCA*ness phenotype, which may not exhibit germline *BRCA* mutations but still demonstrate vulnerabilities such as those with *BRCA* mutations. The clinical application of PARPi has expanded significantly over the past years. Initially approved for breast and ovarian cancers, drugs such as olaparib and rucaparib are now recognized as good treatment options for mCRPC patients with HRR mutations. The TRITON2 study highlighted rucaparib’s efficacy specifically in patients with *BRCA* alterations, while the profound trial demonstrated olaparib’s effectiveness across broader types of HRR mutations [49,50].

Recent studies have also explored combining PARPi with androgen receptor signaling inhibitors (ARSi), such as abiraterone and enzalutamide. These combinations focus on enhancing therapeutic outcomes by targeting multiple pathways involved in tumor growth and survival. For instance, preclinical data suggest that ARSi can induce a functional HRR deficiency, thereby amplifying the effects of PARPi. Clinical trials are ongoing to establish good combinations and sequencing strategies, particularly for patients who may not have identifiable HRR mutations [49,51].

As research continues, several critical questions remain regarding the optimal use of PARPi in PC treatment. Identifying which patient populations will benefit most from these therapies is paramount. Genetic testing for HRR alterations is now recommended for all advanced PC patients to tailor treatment strategies effectively. Additionally, understanding the timing of PARPi administration whether as monotherapy or in combination with other treatments will be essential for maximizing patient outcomes. Furthermore, ongoing studies are investigating the potential use of PARPi in earlier stages of PC treatment, including hormone-sensitive settings. This shift could broaden the applicability of these agents beyond mCRPC and into earlier disease management strategies [48,49,51].

The evolution of chemotherapy in prostate cancer

Recent advancements in chemotherapy for PC have significantly changed treatment paradigms, particularly for mCRPC. As the understanding of the molecular mechanisms underlying PC evolves, new therapeutic strategies and agents are being developed to improve disease outcomes [52].

One of the most promising advances in chemotherapy for mCRPC is the exploration of platinum-based agents, such as carboplatin and cisplatin. Traditionally used in other tumors, these agents are now being investigated for their potential results in PC, particularly in patients with deficient DNA repair mechanisms. Recent studies have shown that platinum therapies can lead to significant PSA response rates ranging from 7.7% to 95%, with overall survival rate improvement reported between 8 and 26.6 months. The unique mechanism of action of platinum compounds, which induce DNA cross-linking and damage, is particularly good for tumors with mutations in DNA repair genes such as *BRCA1* and *BRCA2* [53].

As genetic testing becomes more prevalent in clinical practice, identifying patients with HRR mutations has become crucial for tailoring chemotherapy. Approximately 20% to 25% of men with advanced PC harbor such mutations, making them suitable candidates for targeted therapies such as PARPi. These agents exploit synthetic lethality in HRR-deficient tumors, leading to improved treatment responses. The combination of PARPi with AR pathway inhibitors is currently being explored in clinical trials, aiming to maximize therapeutic benefits for patients who have progressed on standard treatments [49].

Emerging perspectives in zinc transporter research

Zinc transporters, such as the ZIP (Zrt-, Irt-related protein) and ZnTs (zinc transporters), are crucial in regulating zinc concentration in prostate tissues. In healthy prostate cells, these transporters balance zinc flows, which are essential for metabolic processes and the production of prostate fluid. However, in PC, there is a significant dysregulation of these transporters. Specifically, a decline in ZIP transporter expression (e.g., ZIP1, ZIP2, ZIP3) is linked to reduced zinc levels in cancerous tissues. Conversely, certain ZnTs (e.g., ZnT1, ZnT9) show increased expression, further disrupting zinc homeostasis. This dysregulation may cause cancer cells to avoid the cytotoxic effects of high zinc levels. It is suggested that targeting these zinc transporters may offer therapeutic strategies, although the role of zinc supplementation in cancer treatment remains contentious. Some studies suggest potential benefits, while others raise concerns about increased cancer risks with high zinc intake [54].

**Figure 5:**
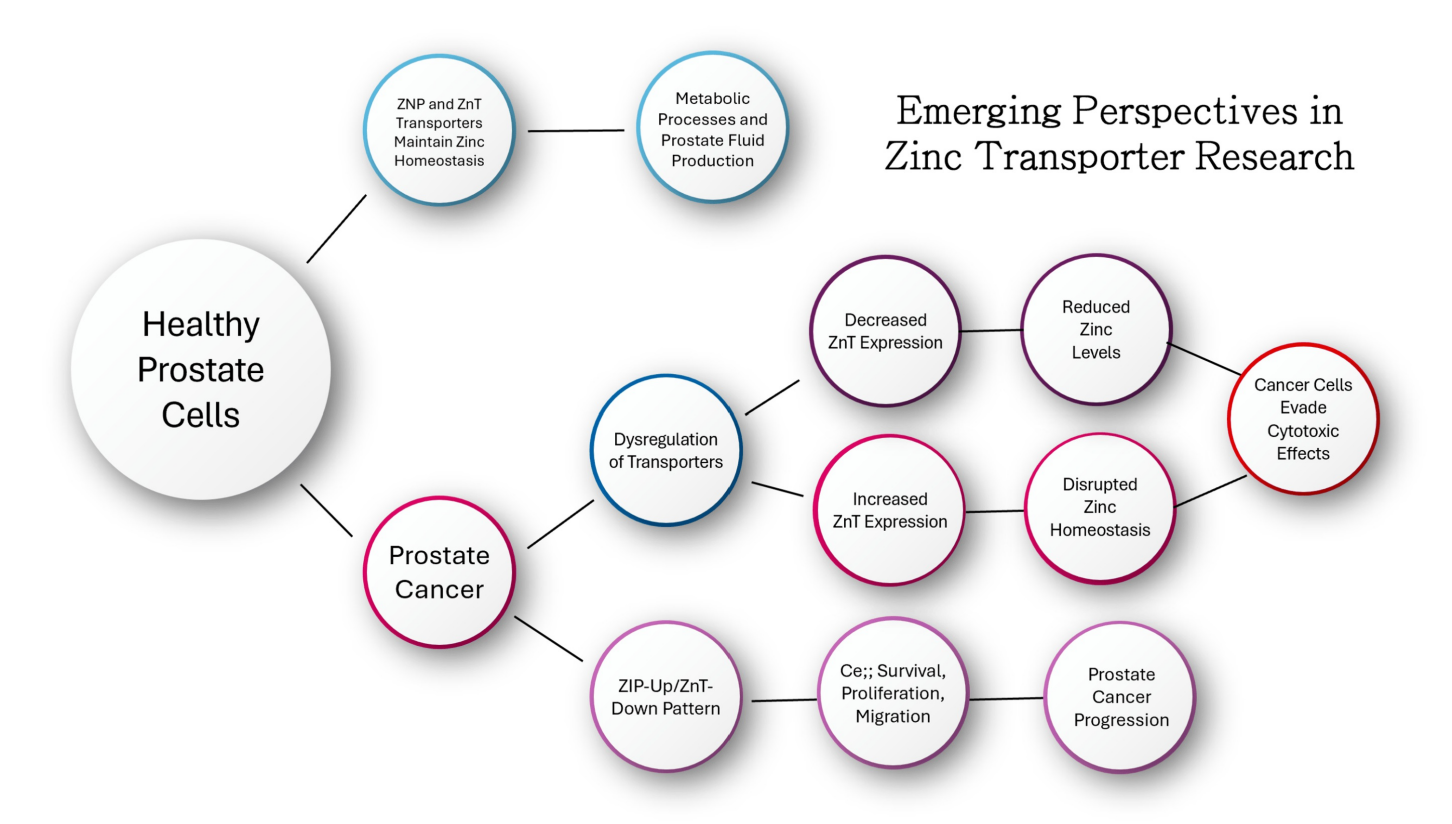
Emerging perspectives in zinc transporter research. Image credit: Reda Harby.

In mouse models and human tumor data, a ZIP-Up/ZnT-Down pattern was identified, where ZIP transporters such as ZIP4 and ZIP8 were upregulated and ZnT2 was downregulated. This pattern, detected in early cancer stages, may play a pivotal role in cancer progression by promoting cell survival, proliferation, and migration. These findings highlight the complexity of zinc’s role in PC, as the molecular variability among tumors suggests that zinc transporter dysregulation differs across individuals. This variability could explain why clinical trials on zinc supplementation yield inconsistent results. Understanding the specific mechanisms by which zinc transporters contribute to tumor growth could improve targeted therapies, especially for patients exhibiting transporter dysregulation profiles [54] (Figure 5).

Focal therapies and minimally invasive treatments in prostate cancer

High-intensity focused ultrasound (HIFU) and cryotherapy have emerged as great alternatives to other treatments. These techniques aim to select only the malignant tissues within the prostate and preserve surrounding normal cells. Recent studies have highlighted the cost-effectiveness and improved quality of life associated with these modalities. For instance, research from Imperial College London indicates that patients undergoing focal therapy experience fewer side effects compared to those receiving invasive treatments, making it a perfect option for many diagnosed with localized PC [55].

High-frequency waves are employed by HIFU to create heat that precisely ablates cells within the prostate. This approach is performed as an outpatient procedure, allowing patients to go home as a same-day case. The technique is particularly good for early-stage PC cases, with reduced collateral damage, which is a common problem with more invasive surgical approaches. Robotic-assisted HIFU enhances the accuracy of the therapy by integrating real-time imaging with prostatic biopsy data, enabling urologists to precisely attack cancer cells. While HIFU has shown good safety and efficacy, some studies indicate a progression rate of up to 37.93% among treated cases, underscoring the need for careful case selection and ongoing monitoring [11,56].

**Figure 6:**
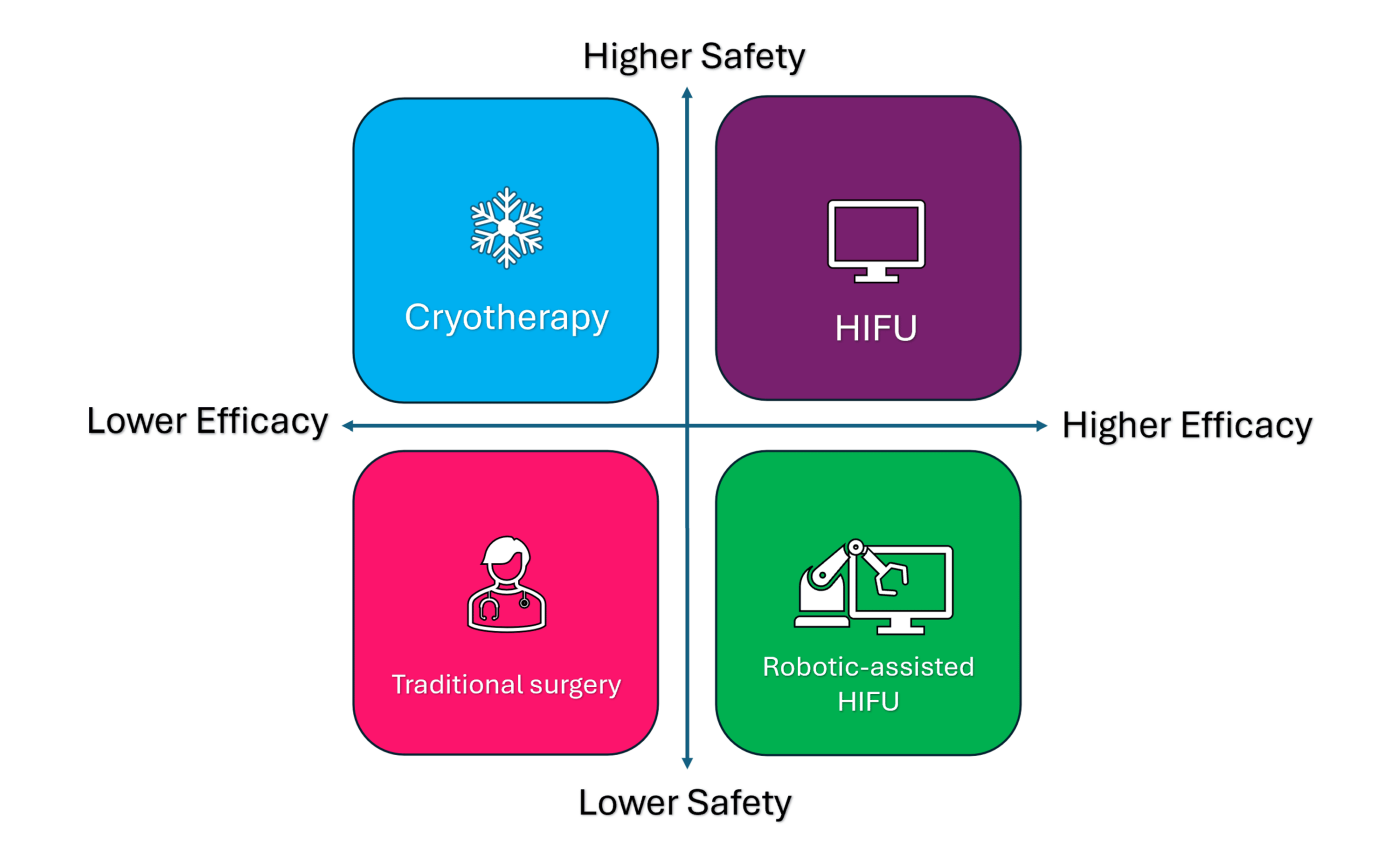
Advantages of focal therapies and minimally invasive treatments. HIFU: high-intensity focused ultrasound Image credit: Momen Abdelglil.

Cryotherapy includes freezing the cells with cell death induction while saving adjacent healthy tissue. This technique has gained a reputation due to its safety profile, especially for patients who may not be ideal candidates for radical surgery. Recent changes in cryotherapy technology have improved its precision through enhanced imaging modalities and temperature control. Studies suggest that cryotherapy can gain high rates of postoperative urinary continence and preservation of sexual function, making it a great approach for many patients. Additionally, long-term results indicate good survival rates and low recurrence rates when appropriately used. As healthcare systems increasingly recognize the value of these minimally invasive approaches, there is a growing push for wider access to focal therapies such as HIFU and cryotherapy in managing PC effectively [56] (Figure 6).

## Conclusions

PC treatment has achieved much progress, particularly in the areas of exactitude medicine, AI immunotherapy, and novel AR-targeted therapies. These revolutions have not only improved the accuracy of diagnostic and therapeutic techniques but also reduced the risks of classic treatments, improving patients’ quality of life. Despite these advances, challenges remain in managing refractory treatment and adjusting individualized treatment. Ongoing research and clinical trials are crucial in reducing and combating these obstacles and refining treatment methods.
